# Model system for the analysis of cell surface expression of human ABCA1

**DOI:** 10.1186/1471-2121-10-93

**Published:** 2009-12-21

**Authors:** Ildikó Kasza, Zoltán Hegyi, Katalin Szabó, Hajnalka Andrikovics, Katalin Német, András Váradi, Balázs Sarkadi, László Homolya

**Affiliations:** 1 Membrane Biology Research Group, Hungarian Academy of Sciences, Department of Biophysics and Radiation Biology, Semmelweis University, Diószegi u 64, H-1113, Budapest, Hungary; 2 Institute of Enzymology, Hungarian Academy of Sciences, Karolina út 29-31, H-1113 Budapest, Hungary; 3 Department of Molecular Cell Biology, National Blood Center, Diószegi u 64, H-1113, Budapest, Hungary

## Abstract

**Background:**

The ABCA1 protein plays a pivotal role in reverse cholesterol transport, by mediating the generation of HDL particles and removing cellular cholesterol. Both the proper expression of ABCA1 in the plasma membrane and the internalization along with apoA-I are required for function. Therefore, we developed a model system to investigate the effect of clinically relevant drugs on the cell surface appearance of ABCA1.

**Results:**

By retroviral transduction system, we established stable mammalian cell lines expressing functional and non-functional ABCA1 variants, tagged with an extracellular hemagglutinin epitope. After characterization of the expression, proper localization and function of different ABCA1 variants, we followed quantitatively their cell surface expression by immunofluorescent staining, using flow cytometry. As expected, we found increased cell surface expression of ABCA1 after treatment with a calpain inhibitor, and observed a strong decrease in plasma membrane ABCA1 expression upon treatment with a trans-Golgi transport inhibitor, Brefeldin A. We tested cholesterol level lowering drugs and other potential inhibitors of ABCA1. Here we demonstrate that ezetimibe affects ABCA1 cell surface expression only in the case of a functional ABCA1.

**Conclusions:**

Our model system allows a quantitative detection of cell surface expression of ABCA1, screening of substrates or specific inhibitors, and investigating transport regulation.

## Background

Elevated low-density lipoprotein (LDL) cholesterol, reduced high-density lipoprotein (HDL) cholesterol, and increased triglyceride levels significantly contribute to the accumulation of lipids in atherosclerotic lesions. Reverse cholesterol transport is believed to be crucial for preventing atherogenesis and hence the development of cardiovascular diseases [1,2]. It is commonly accepted that the ATP-binding cassette protein, ABCA1 plays a pivotal role in the initial steps of the reverse cholesterol transport pathway by mediating the interactions of amphiphilic apolipoproteins (e.g., apoA-I) with cellular lipids to generate nascent HDL particles thus removing excess cellular cholesterol [3,4]. Mutations in ABCA1 cause Tangier disease, a disorder characterized by very low HDL levels, cholesterol deposition in macrophages, and premature atherosclerosis [5-7]. There is increasing evidence suggesting a role for ABCA1 not only in the hepatic but also in the intestinal HDL-production [8].

Therapeutic efforts to raise HDL levels with different drugs have been promising. Clinical trials tested various statins, niacin, and ezetimibe alone and in various combinations to raise plasma HDL- and to reduce the plasma LDL-cholesterol levels [9,10]. Niacin and ezetimibe together with statins were proven to be the most effective combination *in vivo *[11]. However, conflicting results were reported about the effects of statins on lipid efflux and the modulation of ABCA1 expression in *in vitro *experiments [12-16]. Recently, certain types of calcium channel blockers (CCBs), e.g., verapamil, nifedipine, have been found to be anti-atherogenic in clinical trials [17,18]. When their effects on ABCA1 expression were investigated, contradictory results were obtained. These agents either increased ABCA1 mRNA levels or elevated the protein expression without affecting the mRNA level, depending on the cellular test system used [19,20].

Similar to other plasma membrane proteins, the cell surface expression of ABCA1 is modulated by a complex process, which includes transcriptional and post-transcriptional regulation, as well as internalization, degradation, and recycling [21,22]. There is evidence that not only sufficient plasma membrane expression but internalization along with apoA-I is required for proper function of ABCA1 [23,24]. Thus, in the present study we aimed to develop a quantitative *in vitro *test system, which is suitable for monitoring the plasma membrane level of ABCA1, independently from direct transcriptional regulation. In order to generate such an experimental tool, we introduced a hemagglutinin (HA) epitope into the first extracellular loop of ABCA1, and stably expressed this tagged ABCA1 in various mammalian cell lines, using a constitutive promoter. In addition, to make our method suitable for studying trafficking processes of ABCA1 in conjunction with its function, we generated different loss-of-function mutant variants of the HA-tagged transporter. In the present study we demonstrate the applicability and reliability of the developed cellular test system, and report the effects of several pharmaceuticals, which are known to have cholesterol-lowering effects *in vivo*. When studying whether they act through modifying the cell surface expression of functional ABCA1, we found that among several drugs, ezetimibe lowers the plasma membrane level of a functional ABCA1.

## Results

### Stable expression of HA-tagged ABCA1 variants in mammalian cells

**Figure 1:**
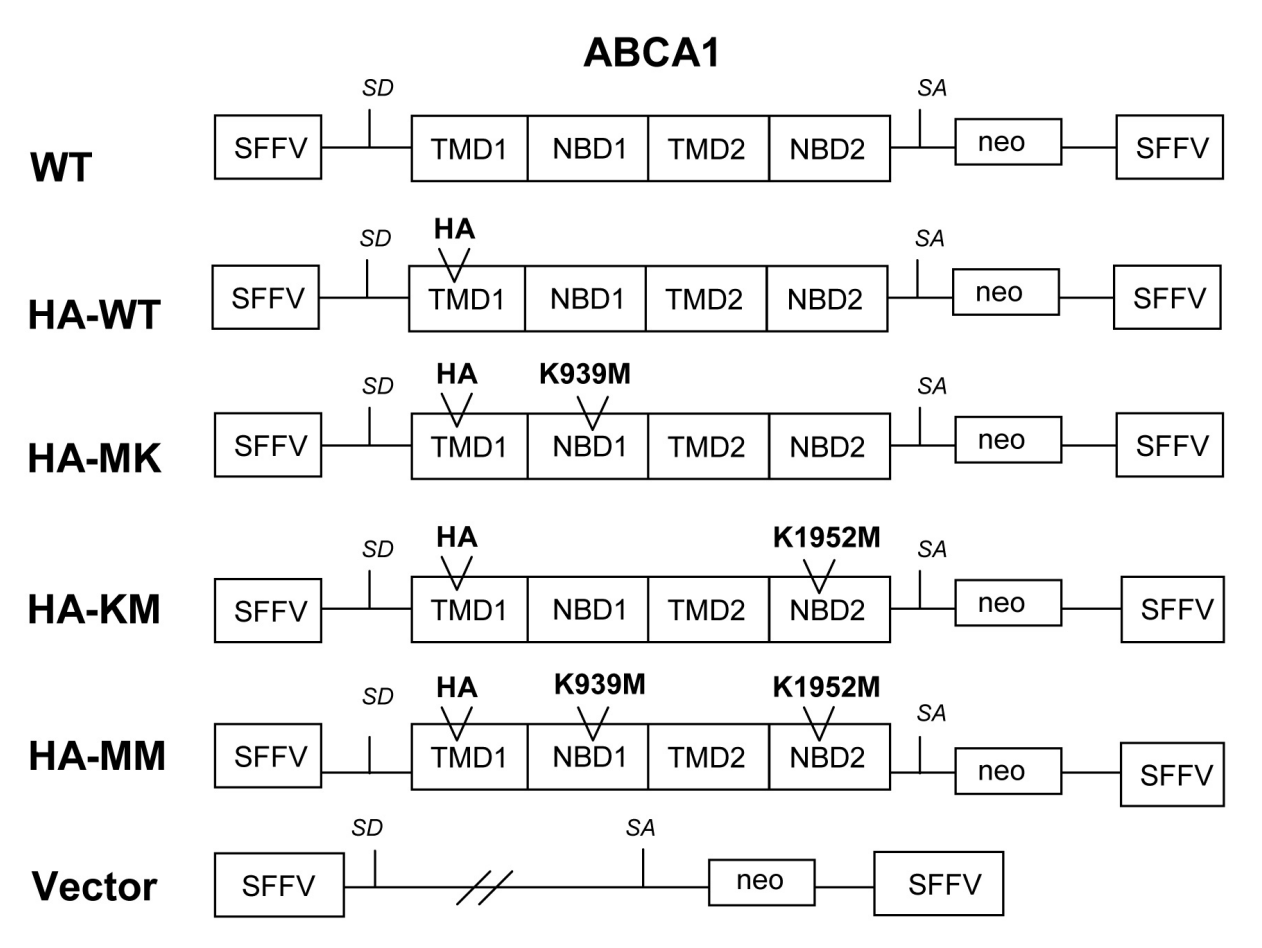
**Schematic representation of bicistronic retroviral vectors**. The ABCA1 cDNA was inserted behind a spleen focus-forming virus (SFFV) retroviral LTR following the splice donor (SD) site. The neomycin resistance (neo) cDNA was inserted after a splice acceptor (SA) site derived from the genome of the Moloney murine leukemia virus. Hemagglutinin (HA) epitope was introduced into the first extracellular loop of the N-terminal transmembrane domain (TMD) of ABCA1 (between residues 207 and 208). WT: wild type untagged ABCA1 construct; HA-WT: wild type HA-tagged ABCA1 construct; HA-MK: a variant with a methionine-lysine substitution (K939 M) in the Walker A motif of the N-terminal nucleotide-binding domain (NBD1); HA-KM: methionine-lysine substitution (K1952 M) in the C-terminal nucleotide-binding domain (NBD2); HA-MM: methionine-lysine substitution in both Walker A motifs (K939 M/K1952 M). A vector containing only the neo cDNA after the SA site was used as a negative control (vector).

Since we aimed to generate different cell lines stably expressing ABCA1 at moderate levels, we used a bicistronic retroviral vector containing ABCA1 and a neo-resistance gene (SsA1neoS) as described in [25]. In order to make our model system suitable for monitoring the cell surface appearance of ABCA1, we introduced an HA epitope into the first extracellular loop of ABCA1 [26]. In addition to the HA-tagged wild type (wt) ABCA1, we generated three mutant variants containing a methionine-lysine substitution in either one or both Walker A motifs (K939 M, K1952 M, and K939 M/K1952 M). These loss-of-function mutations were shown to impair cholesterol efflux and apoA-I binding without affecting protein folding and intracellular routing [27-29]. The assemblies of various retroviral constructs are shown in Figure 1. Retroviral particles were generated by using ecotropic Phoenix and PG13 packaging cells as described in the Materials and Methods section. Thereafter MDCKII and HEK293H cells were transduced and selected with G418 for stable expression of the transgenes [25,30].

**Figure 2:**
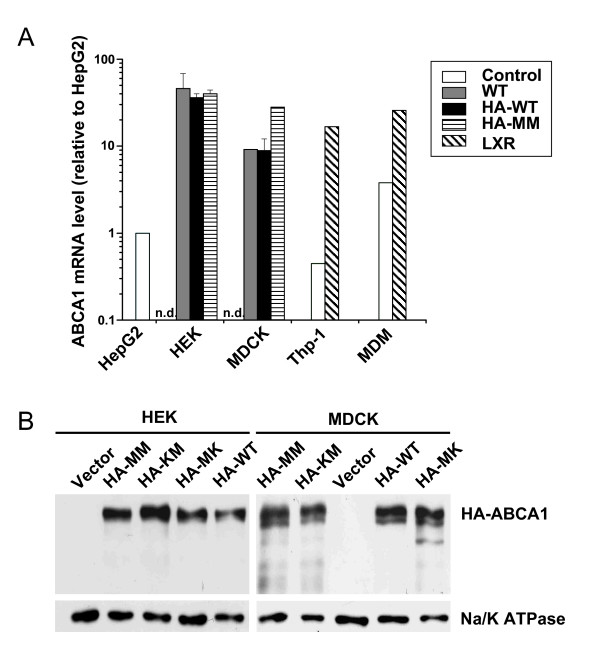
**Expression of wild type and mutant HA-tagged ABCA1 variants in HEK293H and MDCKII cells**. **A**. The mRNA expression level of ABCA1 was measured by real time RT-PCR in the parental HEK293H and MDCKII cells, as well as in cells expressing the ABCA1 variants. The ABCA1 expression of HepG2 cells were used as a reference for relative mRNA expression levels. For comparison, ABCA1 mRNA level was also determined in PMA-pretreated Thp-1 cells and in human monocyte-derived macrophages (MDM). To induce ABCA1 expression, the latter two cell types were treated with 1 μM T0901317, an LXR agonist (LXR). Control: original, parental, or non-treated cells; n.d. - not detected. The expression of ABCA1 variants were similar in the established cell lines and comparable with that seen in LXR-induced macrophages. **B**. The protein expression level of HA-tagged ABCA1 variants was determined using anti-HA antibody by Western blot analysis on whole cell lysates. Panels demonstrate representative blots for the variants of HEK293H and MDCKII cells at passage number 5. Each lane contains 15 μg and 40 μg of protein per well from HEK293H and MDCKII cells, respectively. Anti-Na/K ATPase antibody was used to control the sample loading. For further details see legend for Figure 1.

It has been previously reported that ABCA1 expression rapidly declines in transfected cells after a few passages [31]. In order to obtain a model system with stable expression of each HA-tagged ABCA1 variant at comparable levels, we applied two strategies: i) four different clones with similar expression levels were combined; or ii) the cells, showing similar HA staining, were sorted by FACS. The former method (pooling) was used for the transduced MDCKII cells; whereas the latter (sorting) was used for HEK293H cells. The ABCA1 mRNA expression level of the established cell lines was determined by quantitative RT-PCR, and compared to that of HepG2 cells, macrophage model cells, i.e. PMA-pretreated Thp-1 cells, and human monocyte-derived macrophages (MDM). We found that ABCA1 was moderately over-expressed in our cell lines (app. 10-40-fold as compared to HepG2); nevertheless the expression levels were comparable with that of macrophages induced by the LXR agonist, T0901317 (Figure 2A). The protein expression level in the generated cell lines was also monitored through several passages by Western blot analysis (Figure 2B). Stable transgene expression was found up to 50 and 20 passages in MDCKII and HEK293H cells, respectively.

### Cellular localization and functional characterization of HA-tagged ABCA1 variants

**Figure 3:**
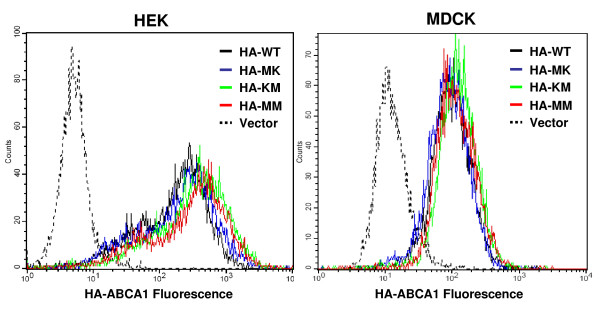
**Flow cytometry analysis of cell surface expression of wild type and mutant HA-tagged ABCA1 variants**. Indirect immunofluorescent labeling with anti-HA antibody and anti-mouse IgG antibody conjugated with Alexa 488 was performed with intact HEK293H and MDCKII cells expressing the different variants of HA-tagged ABCA1. Representative flow cytometry experiments demonstrate the cell surface expression of ABCA1 variants. HA-WT: (black line); HA-MK: (blue line); HA-KM: (green line); HA-MM (red line); vector (dotted line). HA-tagged ABCA1 variants showed comparable plasma membrane appearance in both MDCKII and HEK293H cells.

Besides analyzing the total expression levels, we also studied the cellular localization of ABCA1 variants in the established cell lines. In the first set of experiments indirect immunofluorescent labeling with anti-HA antibody was performed in intact cells, and cell surface staining was detected by flow cytometry. We found comparable plasma membrane expression levels of all ABCA1 variants in both MDCKII and HEK293H cells (Figure 3). Similar to total expression levels of ABCA1 variants, the cell surface expression was also unaffected up to 50 and 20 passages in MDCKII and HEK293H cells, respectively.

**Figure 4:**
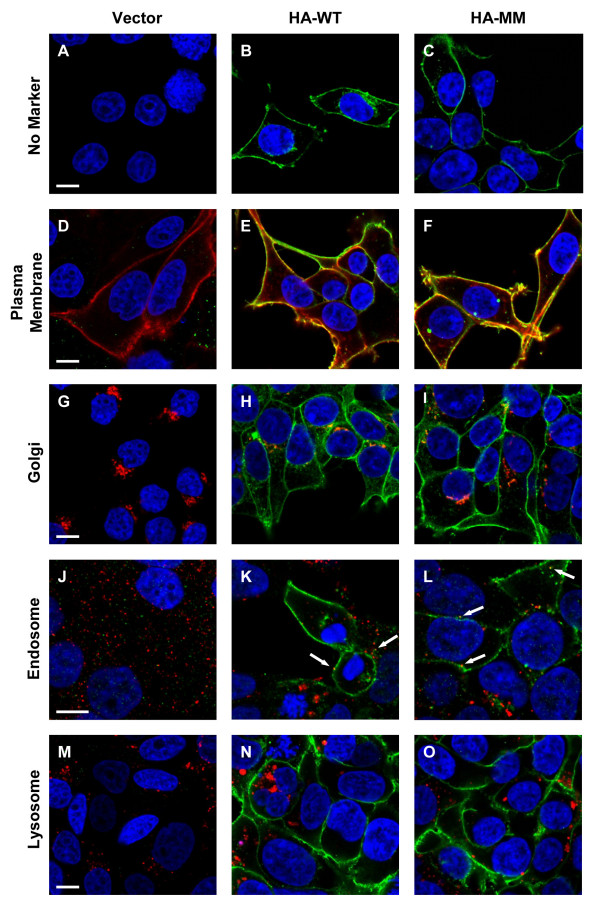
**Subcellular localization of HA-tagged ABCA1 in HEK293H cells**. HEK293H cells expressing the wild type (HA-WT) or double mutant variant (HA-MM) of HA-ABCA1 were immunostained with anti-HA antibody and visualized by confocal microscopy (green). As a negative control HEK293H cells transduced with the empty vector was used. Various cellular markers (wheat germ agglutinin, anti-Giantin and anti-EEA1 antibodies, as well as LysoTracker) were used to identify the different cell organelles (red), whereas DAPI staining was used to label the nuclei (blue). Both wild type and MM variant of HA-ABCA1 were localized predominantly in the plasma membrane. The minor intracellular staining colocalized primarily with the Golgi marker, and rarely with the endosomes (see white arrows). The scale bars indicate 10 μm.

**Figure 5:**
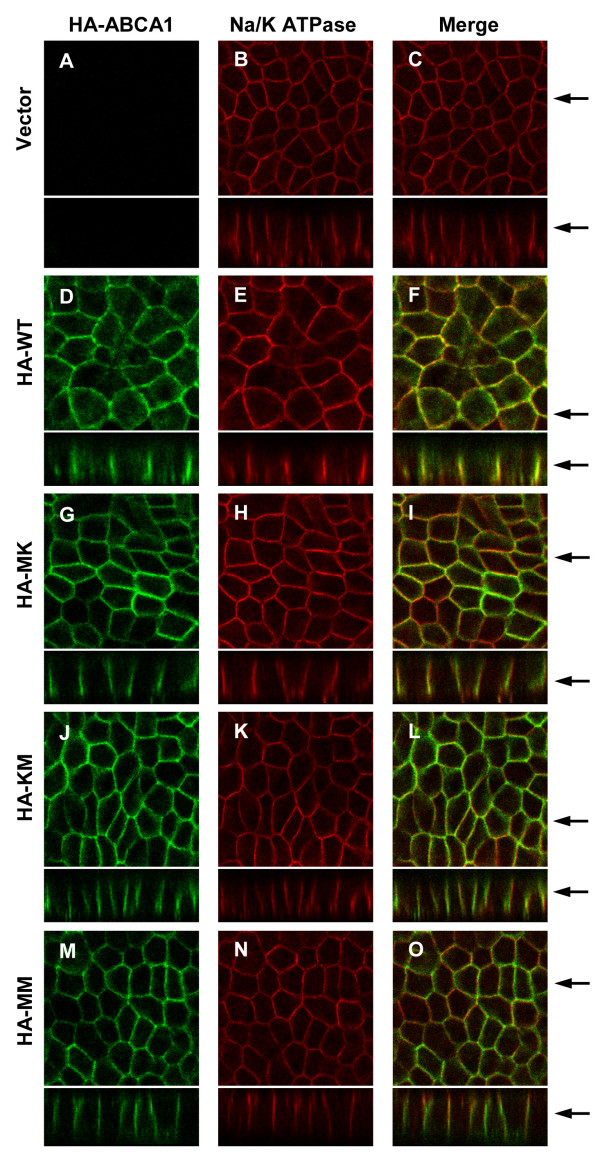
**Subcellular localization of HA-tagged ABCA1 variants in polarized MDCKII cells**. MDCKII cells expressing the wild type (D-F) or mutant variants (G-O) of HA-ABCA1 were grown on a permeable membrane support to form polarized cultures. As a negative control, cells transduced with the neo-containing vector (see Figure 1) were used (A-C). HA-ABCA1 variants were visualized by immunofluorescent staining of permeabilized cells, using confocal microscopy. ABCA1 protein detected by anti-HA antibody is shown in green; staining of Na^+^/K^+ ^ATPase (red) served as a basolateral plasma membrane marker. Right panels depict the overlay. Cross sections are shown at the bottom of each panel; arrows at the side indicate the position of the presented xz and xy planes. All HA-tagged ABCA1 variants exhibited similar and clear basolateral plasma membrane localization, showing only a low level intracellular staining.

The subcellular localization of HA-tagged ABCA1 variants was also studied by immunofluorescent staining followed by confocal microscopy. Detailed subcellular localization analysis performed with HEK293H cells clearly demonstrated that the HA-tagged ABCA1 is expressed predominantly in the plasma membrane (Figure 4E-F). A minor intracellular HA-ABCA1 expression was found in the Golgi-apparatus (Figure 4H-I), and some degree of colocalization was seen with an endosomal marker (Figure 4K-L). The subcellular localization of the HA-tagged ABCA1 variants was also studied in polarized MDCKII. Staining of Na^+^/K^+ ^ATPase was used as a basolateral plasma membrane marker. Similar to previous reports on the untagged protein [32], the HA-tagged wt ABCA1 was also localized to the basolateral membrane compartment (Figure 5D-F), indicating that neither the modest overexpression nor HA-tagging affect proper localization. Similarly, all non-functional HA-tagged ABCA1 variants exhibited basolateral localization (Figure 5G-I, J-L, M-O).

**Figure 6:**
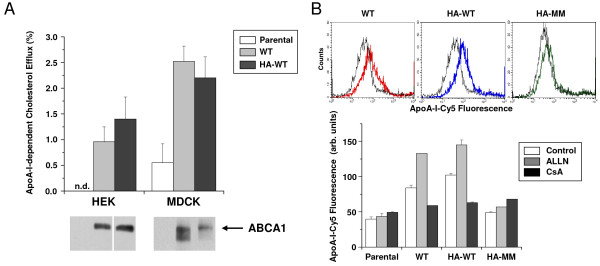
**Functional characterization of the HA-tagged ABCA1**. **A**. The apoA-I-dependent cholesterol efflux was measured in HEK293H and MDCKII cells expressing the untagged (WT) and HA-tagged (HA-WT) ABCA1. As a control the parental cell lines were used. Western blots at the bottom, developed with anti-ABCA1 antibody, indicate the expression levels in the studied cell lines. ABCA1 expression significantly elevated the apoA-I-specific cholesterol efflux in both cell types. HA-tagging did not alter the function of ABCA1. n.d. - not detected. **B**. Binding of Cy5-labeled apoA-I was determined in HEK293H cells expressing the untagged (WT), the HA-tagged wild type ABCA1 (HA-WT) and the mutant form of HA-ABCA1 (HA-MM). Substantial labeling was observed in cells expressing the untagged and the HA-tagged wild type ABCA1 (colored histograms) as compared to parental cells (grey histograms). No apoA-I binding was seen in cells expressing the mutant form of HA-ABCA1. 4 h pretreatment with the calpain inhibitor, ALLN (50 μM) significantly increased apoA-I-binding in both untagged and HA-tagged wt ABCA1-expressing cells, whereas 50 μM cylcosporin A (CsA) pretreatment slightly reduced the apoA-I-binding in these cells. ALLN and CSA had no effect on apoAI-binding of the parental and mutant HA-ABCA1 expressing cells. Control - cells treated with vehicle.

To investigate the functional properties of our HA-tagged ABCA1 variants, we measured the apoA-I-dependent cholesterol efflux and apoA-I binding in the established model cells. In accordance with previous observations with the untagged protein [33,34], ABCA1 expression significantly elevated the apoA-I-specific cholesterol efflux in both HEK293H and MDCKII cells (Figure 6A). More importantly, no difference was observed between the untagged and the HA-tagged ABCA1 in this respect. It is noteworthy that the observed apoA-I-dependent cholesterol efflux was smaller in our case than in preceding reports, most likely due to the moderated ABCA1 expression in our cells versus the massive overexpresssion in the transient expression systems used in previous studies. The apoA-I-dependent cholesterol effluxes from cells expressing the KM, KM, or MM mutants were not significantly different from that seen in the parental cells.

Similar to apoA-I-dependent cholesterol efflux, apoA-I binding was found to be elevated in cells expressing either the untagged or HA-tagged ABCA1, whereas it was unaffected in cells expressing the mutant form of HA-ABCA1 (Figure 6B). Since the calpain inhibitor Acetyl-L-Leucyl-L-Leucyl-L-Norleucinal (ALLN) and cylcosporin A (CsA) were used in further experiments, we investigated the effect of these drugs on the apoA-I-binding in the established cell lines. We found that ALLN significantly increased apoA-I-binding in cells expressing either the untagged or HA-tagged wt ABCA1-expressing, whereas CsA pretreatment slightly reduced the apoA-I-binding in these cells. This observation is in good accordance with previous studies performed with untagged ABCA1 [35-38], and clearly indicates that HA-tagging did not alter the function of the protein. ALLN and CsA had no significant effect on apoAI-binding in the parental cell line and in cell expressing the mutant form of HA-ABCA1.

In summary, we demonstrated reasonable and stable expression, correct subcellular localization, as well as proper function for the HA-tagged ABCA1. Since all three HA-tagged ABCA1 mutants were proven to be non-functional with normal expression and localization, only the wild type HA-tagged and the double mutant variant (MM) were used in further experiments.

### Effect of calpain inhibitor, BFA, and apoA-I on cell surface expression of ABCA1 variants

**Figure 7:**
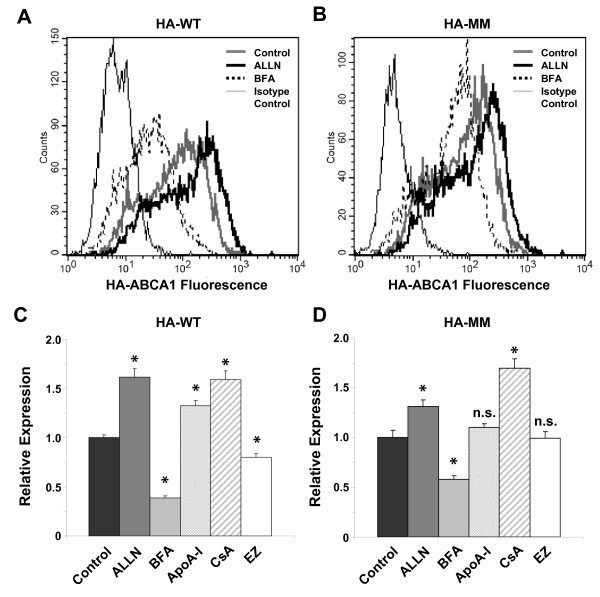
**Effect of different drugs on the cell surface expression of HA-ABCA1 variants**. Flow cytometry analysis of HEK293H cells expressing wild type or MM variant of HA-tagged ABCA1. (A, B) Representative histograms show indirect immunofluorescent staining of intact cells, using anti-HA antibody (thick lines) or mouse IgG1 (isotype control, thin black line). Treatment with 50 μM ALLN (thick black line) substantially increased, whereas 5 mg/ml BFA (dotted line) reduced the cell surface expression level of both wild type (HA-WT) and mutant (HA-MM) HA-ABCA1 variants, as compared to cells treated with vehicle only (control, grey line). (C, D) Cell surface expression of wild type and MM mutant HA-ABCA1 variants in HEK293H cells treated with 50 μM ALLN, 5 mg/ml BFA, 10 μg/ml apoA-I, 10 μM CsA, or 50 μM EZ for 4 h. Relative expression levels were expressed as the geometric mean fluorescence intensities of drug-treated cells normalized to that of vehicle-treated samples (control). Each column represents the mean ± S.E.M. of at least 3 independent experiments. (* p < 0.005; n.s.: non-significant)

In order to demonstrate the applicability of the HA-tagged ABCA1 variants for monitoring cell surface expression, we studied the effect of different substances which are known to influence ABCA1 trafficking and degradation. Since calpain has been shown to participate in ABCA1 degradation [35-37], the HA-ABCA1-expressing cells were treated with the calpain inhibitor, ALLN. This drug was expected to elevate the cell surface expression of ABCA1 due to the inhibition of the degradation pathway. To decrease the cell surface appearance of the protein, Brefeldin A (BFA), an inhibitor of trans-Golgi transport [39] was applied. Indirect immunofluorescent labeling with anti-HA antibody was performed with intact cells 4 h after the treatment with ALLN and BFA, then studied by flow cytometry. Figures 7A and 7B show representative experiments with HEK293H cells expressing wt and MM variant of the HA-tagged ABCA1. Quantitative results are depicted in Figures 7C and 7D. A substantial alteration in the cell surface expression of both variants was observed as compared to non-treated cells. ALLN caused about 1.6-fold increase, whereas BFA treatment resulted in 60% reduction in the plasma membrane level of wt HA-ABCA1 (Figure 7C). It is of note that the effects of drugs were slightly smaller in cells expressing the MM variant of HA-ABCA1 as compared to cells expressing the wt HA-ABCA1 (Figure 7D). Similar to BFA, a general protein synthesis blocker, cycloheximide also reduced the cell surface expression of both ABCA1 variants; however, this drug substantially affected cell viability (data not shown).

ApoA-I has also been demonstrated to increase ABCA1 expression by binding to the transporter and preventing it from degradation [36,37]. As expected, apoA-I significantly increased the cell surface expression of wt HA-tagged ABCA1 (Figure 7C). The MM mutant ABCA1, which was shown to be unable to bind apoA-I [29], exhibited no alteration of the cell surface expression levels when incubated with apoA-I (Figure 7D). These experiments clearly demonstrate that our model system is suitable to detect the effects of various substances on the cell surface expression of either the functional or non-functional variant of ABCA1.

In similar experiments, HA-ABCA1-expressing MDCKII cells were treated with ALLN or BFA. Even though a wide range of drug concentrations was tested, no marked changes in the ABCA1 cell surface expression were seen in these cells. Therefore, in further experiments HEK293H cells expressing the HA-tagged ABCA1 variants were used.

### Effect of different drugs on cell surface expression of ABCA1 variants

By having a verified test system, we studied the effect of various drugs on the cell surface expression of ABCA1 variants. We primarily tested the effect of pharmaceuticals which are known to have cholesterol-lowering effects *in vivo*, to see whether they also act through modifying the cell surface appearance of ABCA1. These drugs included atorvastatin, ezetimibe, and niacin [9], 40-45, as well as calcium channel blockers, nifedipine and verapamil [19,20]. In addition, we investigated the effect of cyclosporin A and glyburide, which have previously been reported to influence the plasma membrane level or function of ABCA1 [29,38]. The studied drugs were applied in a wide concentration range with various incubation times. As positive controls, ALLN and BFA were used to elevate and reduce the ABCA1 cell surface expression, respectively. The results are summarized in Table 1.

**Table 1:** Effect of various substances on the cell surface expression of wild type (HA-WT) and mutant (HA-MM) HA-ABCA1 variants

Drug	Known effect	ABCA1 cell surface expression	
		HA-WT	HA-MM
**ALLN**	Calpain inhibitor	⇧ (+ 60%)	⇧ (+ 30%)

**ApoA-I**	cholesterol acceptor	⇧ (+ 30%)	no effect

**Atorvastatin**	cholesterol level lowering	no effect	no effect

**Brefeldin A**	trans-Golgi transport inhibitor	⇩ (- 60%)	⇩ (- 40%)

**Cycloheximide**	protein synthesis inhibitor	⇩ (- 30%) (viability ⇩)	⇩(- 30%) (viability ⇩)

**Cyclosporin A**	immunosuppressor	⇧ (+ 60%)	⇧ (+ 60%)

**Ezetimibe**	inhibitor of intestinal cholesterol absorption	⇩ (- 20%)	no effect

**Glyburide**	ABCA1 inhibitor	no effect	no effect

**Niacin**	cholesterol level lowering	no effect	no effect

**Nifedipine**	Ca^2+^-channel blocker	no effect	no effect

**Methyl-β-cyclodextrin**	cholesterol depletion	no effect (viability ⇩)	no effect (viability ⇩)

**Cholesterol-methyl-β-cyclodextrin**	cholesterol overloading	no effect (viability ⇧)	no effect (viability ⇧)

**Verapamil**	Ca^2+^-channel blocker	no effect	no effect

We found that atorvastatin (10-50 μM), niacin (0.1-1 mM), verapamil (10-50 μM) and nifedipine (30-100 μM) had no significant effect on the plasma membrane level of ABCA1 even in a wide concentration range. However, CsA (10 μM) substantially elevated the cell surface expression of wt HA-ABCA1 (Figure 7C) in accordance with previous results indicating ABCA1 sequestration in the plasma membrane by CsA [38]. It has not been shown yet whether this effect of CsA is associated with the protein function, therefore we performed similar experiments with the non-functional mutant (MM) variant of ABCA1. We found that CsA equally increased the ABCA1 cell surface expression in wt and MM HA-ABCA1-expressing cells (Figure 7C-D), thus the trapping effect of CsA is independent from ABCA1 function. Unexpectedly, ezetimibe (EZ), a blocker of the intestinal cholesterol absorption, significantly (by 20%) reduced the cell surface expression of wt HA-ABCA1 (Figure 7C). Interestingly, this effect of EZ was found to be associated with ABCA1 function, since no reduction in the cell surface expression of the MM HA-ABCA1 was seen in response to EZ treatment (Figure 7D). Glyburide, which has previously been reported to inhibit ABCA1-mediated cholesterol efflux and apoA-I binding without altering the cell surface levels or total protein levels of ABCA1 [29,46], had no effect of the plasma membrane level of ABCA1 (see Table 1). However, at higher concentrations a slight reduction in the ABCA1 cell surface expression was observed in parallel with a substantial decline in the cell viability in both wt and MM HA-ABCA1-expressing cells. Treatment with 200-500 μM glyburide for 4 h resulted in 50% cell death in both cell types.

We also investigated the effect of plasma membrane cholesterol levels on the cell surface expression of ABCA1 variants. Treatment with 0.1-2.5 mM methyl-β-cyclodextrin (CD), which results in membrane cholesterol depletion [47], had no effect on the plasma membrane level of either wild type or MM variant of HA-ABCA1 even in a wide range of incubation time (20-240 min), although a substantial reduction in the cell viability was observed. Similarly, cholesterol overload by cholesterol-containing CD treatment (0.1-2.5 mM, 20-240 min) did not alter the cell surface expression of ABCA1 in either cell type.

## Discussion

ABCA1 protein is involved in lipid metabolism in several regards. This protein promotes reverse cholesterol transport from the peripheral tissues. ABCA1, being located on the basolateral surface of hepatocytes and intestinal cells, has been shown to play a crucial role in the hepatic and intestinal secretion of HDL [48]. In the present study we generated and characterized a model system for the quantitative assessment of the plasma membrane level of ABCA1. Inclusion of an extracellular hemagglutinin epitope into ABCA1 enabled monitoring of its cell surface expression by flow cytometry and confocal microscopy. By using retroviral transduction we established mammalian cell lines stably expressing functional and non-functional HA-tagged ABCA1 variants. This experimental tool allowed us to investigate the effect of different drugs on cell surface expression of ABCA1.

In previous studies, the protein expression level and subcellular localization of wild type ABCA1 and non-functional mutant forms were found to be identical [27,31]. Similarly, in our model system, both the overall and the plasma membrane expression levels of each HA-tagged ABCA1 variant were comparable, as determined by Western blotting and cell surface labeling (Figures 2B, 3). ABCA1 has been reported to be localized to the basolateral membrane in polarized cells [32]. We found proper subcellular localization for each HA-tagged ABCA1 variant (Figures 4, 5) in accordance with previous reports on the untagged versions of the transporter [27].

Since the applicability and reproducibility of a cellular model system requires stable protein expression levels, we investigated the persistence of our transgene in various cell lines. We found that ABCA1 expression remains stable in MDCKII and HEK293H cells up to 50 and 20 passages, respectively. Thus, our experimental test system substantially surpasses previous attempts to generate cellular models with ABCA1, since in those cases protein expression rapidly declined after a few passages [31]. We demonstrated the applicability of our system to assess the cell surface appearance of ABCA1 by using BFA and ALLN, which have been reported to influence ABCA1 trafficking and degradation (Figure 7A, B).

In agreement with previous reports [35,36], we demonstrated the stabilizing effect of apoA-I on the HA-tagged ABCA1 at the cell surface (Figure 7C). Furthermore, we found that the cell surface levels of the Walker motif mutant ABCA1 (MM), which fails to bind apoA-I (Figure 6B) [28,29], was unaffected by apoA-I (Figure 7D). This observation is in line with previous findings demonstrating that binding of apoA-I is required for the apoA-I-mediated increase of ABCA1 expression [29,37].

In clinical trials, statins were highly effective in reducing LDL-cholesterol, but only moderate effects were seen on the HDL levels. Among a number of different statins, atorvastatin was the most effective and induced redistribution in HDL subpopulations by increasing the fraction of larger HDL particles [49]. Conflicting results were published on the effect of statins on ABCA1 expression and lipid efflux in various cellular systems. Wong et al. demonstrated down-regulation of ABCA1 mRNA and protein levels as well as reduction in the apoA-I-mediated cholesterol efflux in response to statin treatment of PMA-induced Thp-1 cells [14,15]. In contrast, another study found that atorvastatin increased mRNA expression of ABCA1 and promoted cholesterol efflux in the same cell type [12]. In hepatoma cells, statins induced ABCA1 expression resulting in cholesterol and phospholipid efflux to apoA-I [50]. These observations leave the question open, whether statins influence apoA-I-dependent cholesterol efflux solely by changing ABCA1 expression levels or by modifying its subcellular trafficking and cell surface appearance. Since our model system excludes transcriptional regulation, our results revealed that atorvastatin has no effect on the plasma membrane expression of ABCA1.

Niacin has been reported to be one of the most potent agents increasing HDL cholesterol levels [9], and to stimulate ABCA1 expression and function in monocytic cell lines via influencing the cAMP/PKA pathway [51]. By investigating the effect of niacin in our system, we observed no direct effect on cell surface appearance of ABCA1 in accordance with previous observations on the regulatory mechanism of niacin.

Calcium channel blockers have also been reported to influence ABCA1 expression. Hasegawa et al. demonstrated the role of different signaling pathways including PKA, tyrosine kinase, and Janus kinase in ABCA1 expression modulation in response to various CCBs. They found that some of the CCBs, e.g., aranidipine and efonidipine, increase ABCA1 protein expression without altering the mRNA level [20]. In contrast, Suzuki et al. demonstrated a dose-dependent stimulatory effect of other CCBs, such as verapamil, nifedipine, and nicardipine, on both the mRNA and protein expression levels of ABCA1, resulting in elevated cellular lipid release [19]. Our results do not support these findings, since we observed no significant effect on the plasma membrane level of ABCA1 in a wide concentration range of verapamil and nifedipine.

Membrane cholesterol has been implicated to regulate many membrane proteins, including several ABC transporters, e.g. ABCB1 and ABCG2 [47,52,53]. Membrane microdomains with high cholesterol content have been suggested to play a role in such regulation [47]. However, neither cholesterol overload nor depletion influenced ABCA1 cell surface expression in our model system. It is noteworthy that treatment with methyl-β-cyclodextrin (CD) reduced, whereas cholesterol-loaded CD significantly increased cell viability. The most plausible explanation for this observation is that CD treatment makes the cell membrane fragile and more sensitive to mechanical stress. This notion should be taken in account in future studies on ABCA1-expressing cells treated with CD.

We studied the effect of glyburide and CsA, which have previously been reported to influence the plasma membrane level or function of ABCA1 [29,38]. In accordance with previous observations, glyburide had no effect on ABCA1 plasma membrane appearance in our model system [29], whereas CsA greatly increased the cell surface level of ABCA1 (Figure 7C). ALLN elevated both ABCA1 cell surface expression and apoA-I binding, whereas CsA reduced apoA-I binding despite the increased plasma membrane level of ABCA1. This observation is line with a previous report suggesting that CsA sequesters ABCA1 in the plasma membrane [38]. The observation that cell surface expression of the non-functional ABCA1 mutant (MM) is also increased by CsA pretreatment clearly indicates that the trapping effect of this drug is independent from ABCA1 function.

Ezetimibe, a drug which is known to block the intestinal cholesterol absorption, has not only an inhibitory effect on several brush border membrane proteins, including Niemann-Pick C1-like 1, scavenger receptor B-I and CD36 [54], but has also been reported to lower the mRNA level of several other genes involved in lipid metabolism. The transcription of ABCA1 is also reduced by EZ [55], but no study has yet been reported on its effect on the cell surface expression of ABCA1. Our model system allows us to investigate the plasma membrane expression of ABCA1 independently from its transcriptional regulation. Strikingly, we found that EZ significantly lowers the cell surface expression of wt HA-ABCA1 (Figure 7C). Since no reduction in the cell surface expression of the MM HA-ABCA1 was seen in response to EZ treatment (Figure 7D), the effect of EZ occurs only with a functional ABCA1, thus, its effect is likely connected to a functional form of the protein.

There are several possibilities to explain the lack of effect of EZ on cell surface expression of the non-functional MM-ABCA1 variant. It is theoretically possible but very unlikely that EZ inhibits the trafficking of only the *de novo *synthesized functional proteins. It is more plausible that EZ influences the protein internalization and recycling to the plasma membrane, since ABCA1 has been shown to undergo a relatively rapid internalization and recycling to the plasma membrane, which process is widely accepted to require protein function [39,56]. Therefore, if EZ either accelerates protein internalization or inhibits recycling, and in the meantime MM-ABCA1 has a lower internalization and recycling turnover compared to wild type, EZ causes a smaller effect on the cell surface appearance of the mutant transporter. This hypothesis is supported by the observation that the effects of ALLN were also smaller in HA-MM-ABCA1-expressing cells, as compared to wild type HA-ABCA1. It is also possible that EZ accelerates ABCA1 degradation, since the internalization itself is dependent on the transporter function. This idea is not supported by our recent observations using confocal microscopy, when we found that following EZ treatment the wt HA-ABCA1, but not the MM variant, accumulated intracellularly in vesicles but not in lysosomes (data not shown). Nevertheless, further studies are needed to reveal the mechanism of EZ effect on the cell surface expression of ABCA1.

## Conclusions

In the current study, we have provided a model system suitable for the quantitative assessment of the plasma membrane level of ABCA1. We have shown that this test system can be reliably applied for studying the effect of various pharmaceuticals on the cell surface expression of the transporter. Therefore, our model system provides a new tool for acquiring more information on the post-translational regulation, internalization, degradation and recycling of the ABCA1 protein.

## Methods

### Plasmid constructs

For the generation of SsA1neoS vector, the full length wild type human ABCA1 cDNA [57] was cloned into a bicistronic retroviral SPsLdS vector [58], which was modified to contain the neomycin resistance cassette (neo) as previously described [25]. A cassette containing the coding sequence of an HA epitope (YPYDVPDY) was inserted into the full-length human ABCA1 cDNA, between residues 207 and 208 [26]. For control experiments, a vector containing only the neo cDNA was generated. The HA-ABCA1 K939 M, K1952 M mutants were constructed in the pBlscrSK- vector containing XhoI/NotI cassette from the SsHAA1neoS vector. HA-ABCA1 K939 M was constructed by the overlap extension PCR technique using the internal 5'-TGGCTGTGATCATCAAGGGC-3' and 5'-CCAGGACGTCCGCTTCATCCAT-3' and external 5'-ATTGACATGGTGGTCGTC**A**TCC-3' and 5'-GGA**T**GACGACCACCATGTCAAT-3' oligonucleotides. The external primer pair includes AAG (lysine) to ATG (methionine) single mutation and a new FokI restriction site (in bold). The HA-ABCA1 K1952 M mutant was generated by using 5'-AGCCAAAAGCTTAAAGAACAAGATCTGGGTG-3' and 5'-CTCCTGTTAACATCTTGAAAGTTGATGA**CA**TCCCAGCC-3' oligonucleotides, which results in a lysine to methionine mutation (in bold) and contains a GC substitution (underlined) to create a new FokI restriction site. The mutant constructs were verified by FokI digestion. HA-ABCA1 variant containing double methionine substitution was created by cassette exchange in pBlscrSK- vector by using HindIII/HpaI digestion. After partial digestion of each mutant construct in pBlscrSK- vector by XhoI/BamHI enzymes the fragments were transferred into SsHAA1neoS vector and verified by sequencing.

### Cell culturing

Phoenix-eco [59] was a gift from G. Nolan (Department of Pharmacology, Stanford University, Stanford, CA); PG13 retrovirus producing cells [60], HepG2, Thp-1, and MDCKII cells were obtained form the ATCC (Manassas, VA). HEK293H cells were purchased from Invitrogen. Most cell lines were grown in DMEM containing 10% FCS, whereas HepG2 were cultured in DMEM and F12 (1:1) + 10% FCS medium. Thp-1 cells were maintained in RPMI + 10% FCS medium, and pretreated with 2 nM PMA for 24 h. Human monocytes were isolated from peripheral blood of healthy subjects, as previously described [61], and cultured in DMEM containing 10% FCS for 5 days. ABCA1 expression was induced in both PMA-pretreated Thp-1 cells and monocyte-derived macrophages by the addition of a synthetic LXR agonist, T0901317 (1 μM, Alexis Biochemicals) 8 h prior to studies.

### Generation of cell lines stably expressing ABCA1 variants

Retroviral particles and cell lines stably expressing the ABCA1 variants were generated as described previously [25,30] with some modifications. Briefly, the Phoenix-eco packaging cell line was transfected by calcium phosphate coprecipitation (Invitrogen). The cell-free viral supernatant was collected 24 and 48 hours after transfection and immediately used to transduce PG13 cells. The transduction of PG13 cells was repeated on the following day. HEK293H and MDCKII cells were transduced with viral supernatant collected from PG13 packaging cells 24 and 48 hours after transduction. The target cells were then selected with 0.5 mg/ml and 0.8 mg/ml G418 for HEK293H and MDCKII cells, respectively. Single-cell clones of transduced MDCKII cells were isolated by limiting dilutions on 96-well plates under continuous G418 selection. In contrast, HEK293H cells were labeled with direct anti-HA antibody and sorted by a FACSAria (Becton Dickinson) cell sorter. To reduce the heterogeneity, a subsequent cell sorting was performed.

### RNA isolation and real time RT-PCR

Total RNA was isolated by a single-step method using TriZol (Invitrogen). The purity of the RNA preparation was checked by measuring the absorbance ratio at 260/280 nm. For each reaction 1 μg total RNA was reverse-transcribed into cDNA according to the manufacturer's protocol (Promega) using random hexamer oligonucleotides. The resulting cDNA was then subjected to real-time quantitative PCR (Light Cycler 480 II, Roche), 45 cycles of 95°C for 30 s, 58°C for 1 s and 72°C for 1 s by using specific primers to detect human ABCA1 cDNA, but not the genomic *ABCA1 *sequence. Oligonucleotides designed for PCR analysis of ABCA1 or housekeeping Glyceraldehyde 3-phosphate dehydrogenase (GAPDH) cDNAs were: 5-ACAAGATGCTGAGGGCTGAT-3 and 5-CCCAAGACTATGCAGCAATG-3 or 5-GAAGGTGAAGGTCGGAGTCA-3 and 5-GACAAGCTTCCCGTTCTCAG-3, respectively. PCRs contained 3 mM MgCl_2_, 0.25 μM from ABCA1 and 0.5 μM from GAPDH forward and reverse primers, and 5 μl LightCycler DNA Master SYBR Green I (2×, Roche). Before amplification, a preincubation step (60 seconds at 95°C) was performed to activate FastStart DNA polymer and to ensure complete denaturation of the cDNA. Following the last cycle, melting curve analysis was carried out to specify the integrity and purity of the amplicons. Amplification profiles were analyzed using the Fit Points and 2nd Derivative Maximum calculation described in the LightCycler Relative Quantification Software. The comparative Ct method was used to quantify transcript levels and to normalize for GAPDH expression.

### Western blot analysis

Preparation of whole cell lysates and immunoblotting were performed as described previously [62] with some modifications. Cell were suspended in 2× Laemmli buffer and sonicated for 5 seconds at 4°C. 15 μg and 40 μg of protein per well from HEK293H cells and MDCKII cells, respectively, were loaded onto a Laemmli-type 6% SDS-polyacrylamide gel, separated, and blotted onto PVDF membranes. Immunodetection was performed by using an anti-HA monoclonal (HA.11, BabCo, 1:3000) or rabbit polyclonal anti-ABCA1 (Abcam, 1:250) antibodies. A HRP-conjugated goat anti-mouse or anti-rabbit IgG secondary antibody (Jackson Immunoresearch, 1:10.000), and enhanced chemiluminescence (ECL) technique was applied. For loading control, a mouse anti-Na^+^/K^+ ^ATPase antibody (BioMol, 1:1000) was used.

### Confocal microscopy

For immunolocalization studies, HEK293H cells were seeded onto eight-well Lab-Tek II Chambered Coverglass (Nalge Nunc) at 5 × 10^4 ^per well cell density, and grown for 2 days. For plasma membrane staining, the cells were gently fixed with 1% paraformaldehyde (PFA) for 5 min at room temperature, and incubated with 5 μg/ml AlexaFluor633-conjugated wheat germ agglutinin (WGA, Invitrogen) for 10 min at room temperature. For lysosome staining, the living cells were subjected to LysoTracker Red (Invitrogen) in culturing medium for 30 min at 37°C. After marker staining the cells were fixed and permeabilized with 8% PFA for 15 min at room temperature, and labeled with anti-HA (HA.11, MMS-101R BabCo, 1:500) antibody. To label the Golgi-apparatus and endosomes, double-labeling was performed using rabbit anti-Giantin (Abcam, 1:500) and anti-EEA1 antibodies (1:250), respectively, as described previously [61]. For secondary antibodies AlexaFluor-488-conjugated anti-mouse IgG and AlexaFluor-594-conjugated anti-rabbit IgG antibodies (Invitrogen, 1:250) were used. After immunolabeling the samples were counterstained with 1 μM DAPI for 10 min at room temperature.

For subcellular localization studies in polarized cells, the MDCKII cells were seeded at 5 × 10^5 ^cells/cm^2 ^density on 6.5-mm Transwell Col filters (pore diameter 0.4, Corning Costar) previously coated with 0.03 mg/ml Vitrogen (Cohesion Technologies). The cultures were regularly washed with culturing medium to remove cell debris. Studies were carried out with confluent cultures 10 days after seeding. The cultures were permeabilized and labeled with anti-HA (HA.11, MMS-101R BabCo, 1:500) and anti-Na^+^/K^+ ^ATPase (Abcam, 1:250) antibodies, as previously described [61]. AlexaFluor-488-conjugated anti-mouse IgG and AlexaFluor-647-conjugated anti-chicken IgG secondary antibodies (Invitrogen, 1:250) were used.

The blue, green, red and far red fluorescence of stained samples was studied by an Olympus FV500-IX confocal laser scanning microscope using a PLAPO 60× (1.4) oil immersion objective (Olympus) at 405, 488, 543, and 633 nm excitations.

### Cholesterol efflux measurement

Cholesterol efflux measurement was performed as described previously [34]. Briefly, near confluent HEK293H and non-polarized MDCKII cultures grown on 6 well plates were labeled with 100 nM (4 μCi/ml) ^3^H-cholesterol (PerkinElmer) at 37°C for 24 hours in serum-free DMEM containing 2 mg/ml fatty acid free BSA. The media were then replaced with fresh DMEM/BSA in the presence or absence of 15 μg/ml of apoA-I and incubated at 37°C for 24 hours. Supernatants were collected and spun down to remove cellular material. The pellets and the cell layers were lysed in 2% SDS and 1 M NaOH. The radioactivity of both samples was measured by liquid scintillation; the cholesterol efflux was expressed as the percentage of total (extruded plus cellular) ^3^H-cholesterol. The apoA-I-dependent efflux was obtained from the difference of the effluxes measured in the presence and absence of apoA-I.

### Cell surface expression studies

For cell surface expression studies, the cells were detached by trypsinization and after washing steps blocked for 10 min at room temperature in phosphate-buffered saline containing 2 mg/ml bovine serum albumin (BSA/PBS). The samples were then incubated for 40 min at room temperature with anti-HA antibody (HA.11, MMS-101R BabCo, 1:250 in BSA/PBS). After subsequent washing steps AlexaFluor-488-conjugated anti-mouse IgG secondary antibody (Invitrogen, 1:500 in BSA/PBS) was applied for 40 min at room temperature. As an isotype control a mouse IgG1 (1:10) from Sigma-Aldrich was used. The cell surface expression of the HA-ABCA1 variants was detected by flow cytometry (Becton Dickinson FACS Calibur).

When the modifying effect of various drugs was studied, the cells in the culture dish were pretreated for 4 hours with the following substances: 50 μM ALLN, 10 μg/ml apoA-I (Calbiochem), 10-50 μM atorvastatin (Pfizer), 5 μg/ml BFA, 10-50 μM ezetimibe (Schering-Plough), 0.1-1 mM niacin, 10-50 μM verapamil, 30-100 μM nifedipine, 50-500 μM glyburide, 10-50 μM cyclosporin A, 100 μg/ml cycloheximide, 0.1-2.5 mM random methylated beta cyclodextrin (CD, CycloLab), or 0.1-2.5 mM CD preloaded with 4.4% cholesterol (CycloLab). The materials were obtained from Sigma-Aldrich if not indicated otherwise. After treatment, cells were trypsinized, pre-stained for 2 min with 10 mg/ml propidium-iodide, and gently fixed with PFA for 10 min. Immunofluorescent staining of drug-treated cells was performed at 4 C. Relative expression levels were expressed as the ratio of geometric mean fluorescence of drug-treated and the vehicle-treated samples. For statistical analysis Student's t test was used.

### ApoA-I binding

ApoA-I conjugation and binding-experiments were performed as described previously [28] with minor modifications. Briefly, apoA-I was conjugated to the fluorochrome Cy5 (PA25001, Amersham Pharmacia Biotech) according to the recommendation of the manufacturer. For all experiments the labeled apoA-I (apoA-I-Cy5) was diluted in binding buffer (1.8 mM CaCl_2_, 1 mM MgCl_2_, 5 mM KCl, 150 mM NaCl, 10 mM HEPES, pH 7.4), and aggregates were removed by ultracentrifugation for 30 min at 100,000 g. Binding was performed in the presence of 20 μg/ml of apoA-I-Cy5 for 1 h at 4°C on 5 × 10^5 ^cells detached by trypsinization. After the incubation, cells were rapidly washed and not fixed with 1% PFA. For studying the effect of ALLN and CsA, the cells were pretreated with same concentrations of drugs as in the cell surface expression studies. After incubation for 4 hours cells were washed and apoA-I binding experiment was performed. The apoA-I binding was detected by flow cytometry (Becton Dickinson FACS Calibur) and analyzed by CellQuest Pro software. Binding data are calculated from the median of apoA-I-Cy5 fluorescence intensity.

## Authors' contributions

IK participated in generation of HA-ABCA1 variant plasmid constructs and retroviral transduction processes of HEK293H cells; carried out characterization of cell lines and cell surface expression studies by flow cytometry; as well as contributed to manuscript preparation. ZH participated in generation of plasmid constructs; prepared MDCKII cell lines expressing HA-ABCA1 variants; carried out the Western blot analysis and cholesterol efflux measurements. KSZ coordinated the retroviral transduction process, immunofluorescent labeling, and flow cytometry studies; generated plasmids and performed cholesterol efflux measurements. HA participated in design of plasmid constructs, carried out plasmid sequencing. KN participated in design and coordination of the retroviral transduction work. AV and BS participated in design and coordination of the study and helped in manuscript preparation. LH contributed to the design and coordination of the study, coordinated immunofluorescent staining and carried out confocal microscopy studies, as well as participated in manuscript preparation.
